# Biomarkers of endothelial damage in erectile dysfunction based on preclinical studies: a systematic review

**DOI:** 10.1007/s11255-025-04895-3

**Published:** 2025-11-21

**Authors:** María Paula Gómez-Bueno, Marcelo Marconi, Herney Andres Garcia-Perdomo

**Affiliations:** 1 UROGIV Research Group, School of Medicine, Universidad del Valle, Cali, Colombia; 2 Division of Urology/Urooncology, Department of Surgery, School of Medicine, Universidad del Valle, Cali, Colombia; 3 Urology Department, Pontificia Universidad Católica de Chile, Santiago, Chile

**Keywords:** Biomarkers, Endothelial progenitor cells, Endothelium, Erectile dysfunction

## Abstract

**Purpose:**

To determine the relationship between biomarkers and endothelial damage in animal models of experimentally induced erectile dysfunction. The biomarkers with the most robust evidence could be tested in future in human patients to assess their clinical significance in the diagnosis and follow-up of ED patients.

**Methods:**

This systematic review was conducted according to the recommendations of the Cochrane Collaboration and following the PRISMA Statement. We designed a search strategy in MEDLINE (OVID), EMBASE, and the Cochrane Central Register of Controlled Trials (CENTRAL) from inception to the present. We assessed the risk of bias based on the SYRCLE tool for preclinical trials.

**Results:**

1208 animals were included in 34 studies. A total of 12 biomarkers were reviewed. Six markers showed decreased expression: eNOS in 18 studies (*p* < 0.05), in 9 studies (*p* < 0.01), and one study (*p* > 0.05); VEGF in 5 studies (*p* < 0.05) and one study (*p* < 0.01); nNOS in 1 study (*p* < 0.01) and in 1 study (*p* < 0.05); aSMA in 2 studies (*p* < 0.05); LncRNA in 1 study (*p* < 0.05); Hebp1 in 1 study (*p* < 0.001). Six markers showed increased expression: FACL-4, PACS-2, and IP3R1 in 1 study (*p* < 0.01, respectively); endocan in 1 study (*p* < 0.05); endothelial microparticles and endothelial progenitor cells in 1 study (*p* < 0.05, respectively). The six markers with decreased expression showed more than a 50% reduction compared to the control group. The six markers that showed increased expression demonstrated an increase of more than 50% compared to their respective comparison group. Selection, performance, and detection biases were identified in 100% of the studies.

**Conclusion:**

eNOS; VEGF; nNOS; aSMA; LncRNA; and Hebp1 showed decreased expression in the presence of endothelial damage, while FACL-4; PACS-2; IP3R1; endocan; endothelial microparticles; and endothelial progenitor cells showed an increase under the same conditions. These could become markers with implications for clinical trials and the development of new targeted treatments for ED.

## Introduction

Erectile dysfunction (ED) is often linked to endothelial dysfunction, which can be a precursor to cardiovascular diseases. Biomarkers play a crucial role in diagnosing and managing ED. The role of increased expression of endocan in mice with low testosterone states leads to inhibition of the AKT/eNOS/NO signaling pathway. This inhibition results in impaired erectile function. Endocan, also called endothelial cell-specific molecule 1 (ESM-1), is a soluble dermatan sulfate proteoglycan, as a novel biomarker, indicating the bioactivity of endothelial cells [1, 2]. Additionally, low androgen levels lead to increased expression of fatty acid-CoA ligase 4 (FACL-4), Phosphofurin Acidic Cluster Sorting Protein 2 (PACS-2), inositol 1,4,5-trisphosphate receptor type 1 (IP3R1) in the mitochondria-associated membranes of the rat penile corpus cavernosum [3].

Nitric oxide (NO) has vasodilatory properties; endothelial NO synthase (eNOS) and neuronal NO synthase (nNOS) isoforms are tightly regulated and produce physiologically relevant levels of NO in endothelial cells and autonomic nerve endings of the penis [4]. α-Smooth muscle actin (ASMA) is an accepted marker of smooth muscle cells (SMC) in the corpora cavernosa, which usually decreases corporal veno-occlusive dysfunction [5]. Heme-binding protein 1 (Hebp1) is an intracellular tetrapyrrole-binding protein possibly involved in heme or porphyrin biosynthesis. Hebp1 promotes neurovascular regeneration in diabetes-induced ED by restoring endothelial and neural cell contents and reducing reactive oxygen species levels [6]. Vascular endothelial growth factor (VEGF) is a multifunctional protein that plays a role in angiogenesis stimulation and apoptosis inhibition [7]. Long noncoding RNA – myocardial infarction-associated transcript (LncRNA-MIAT) knockdown could further decrease viable cells, reduce cell viability, accelerate cell apoptosis, and inhibit cell proliferation [8].

Extracellular vesicles from endothelial cells are key to cellular communication. This expression is lower in rat organisms that have not been exposed to androgens; therefore, signaling molecules affecting vascular endothelial function regulation could be lost [9]. Endothelial microparticles (EMPs) increase in conditions like hypertension; they can impair endothelial function by reducing nitric oxide production and promoting vascular inflammation. Endothelial progenitor cells (EPCs) are crucial for vascular repair and regeneration; a lower ratio of EPCs indicates a negative balance between endothelial damage and repair [10].

We aimed to determine the relationship between biomarkers and endothelial damage in animal models of experimentally induced erectile dysfunction. The biomarkers with the most robust evidence could be tested in future in human patients to assess their clinical significance in the diagnosis and follow-up of ED patients.

## Methods

We conducted this systematic review according to the recommendations of the Cochrane Collaboration and following the PRISMA Statement.

### Eligibility criteria

*Study designs*: We included preclinical studies.

*Participants*: Animal models (rats or mice) with experimentally induced erectile dysfunction.

*Intervention*: Evaluation of specific biomarkers of endothelial damage (FACL-4, PACS-2, IP3R1, eNOS, nNOS, aSMA, Hebp1, VEGF, lncRNA MIAT, endothelial microparticles, endothelial progenitor cells, and endocan), at least five studies involving five animals are included to ensure statistically significant results.

*Comparison*: Healthy animal models or models of erectile dysfunction without specific intervention.

*Primary outcome*: Identification of biomarkers significantly associated with endothelial damage and erectile dysfunction in preclinical studies.

### Exclusion criteria

Population is less than five animals.

### Information sources and search strategy

We designed a search strategy in MEDLINE (OVID), EMBASE, and the Cochrane Central Register of Controlled Trials (CENTRAL) from inception to the present (Appendix 1). To ensure literature saturation, we scanned references from relevant articles identified through the search, conferences, thesis databases, Open Grey, Google Scholar, and others. We contacted the authors by e-mail in case of missing information. No language or restrictions were imposed. The search was conducted between March and July 2025.

### Data collection

Two researchers reviewed each reference by title and abstract. Then, reviewers confirmed all data in full texts of relevant studies, applied pre-specified inclusion and exclusion criteria and extracted the data. Disagreements were resolved by consensus, and where disagreement could not be solved, a third reviewer resolved the conflict.

Relevant data were collected using a standardized form to extract the following information from each article: study design, geographic area, species of study animal, number of individuals, biomarker, extraction tissue, biomarker detection methodology, results according to decrease or increase expression in endothelial damage, and statistical significance.

### Risk of bias

We assessed the risk of bias using the SYRCLE (Systematic Review Centre for Laboratory Animal Experimentation) tool. The questions are distributed according to bias as follows: Selection (question 1–3); Performance (question 4–5); Detection (question 6–7); Attrition (question 8); Reporting (question 9); Other (question 10).

### Data analysis/synthesis of results

We could not perform a meta-analysis due to the high clinical and methodological heterogeneity.

### Statistical analysis

Continuous quantitative variables (number of individuals) were analyzed, and results were presented in percentages to facilitate data interpretation and comparison. Data are expressed as relative expression rates in the control or intervention group. The proportion of decrease or increase in the marker is expressed as *X* = relative rate (intervention) * 100 / relative rate (control)—100%.

## Results

### Study selection

**Fig. 1:**
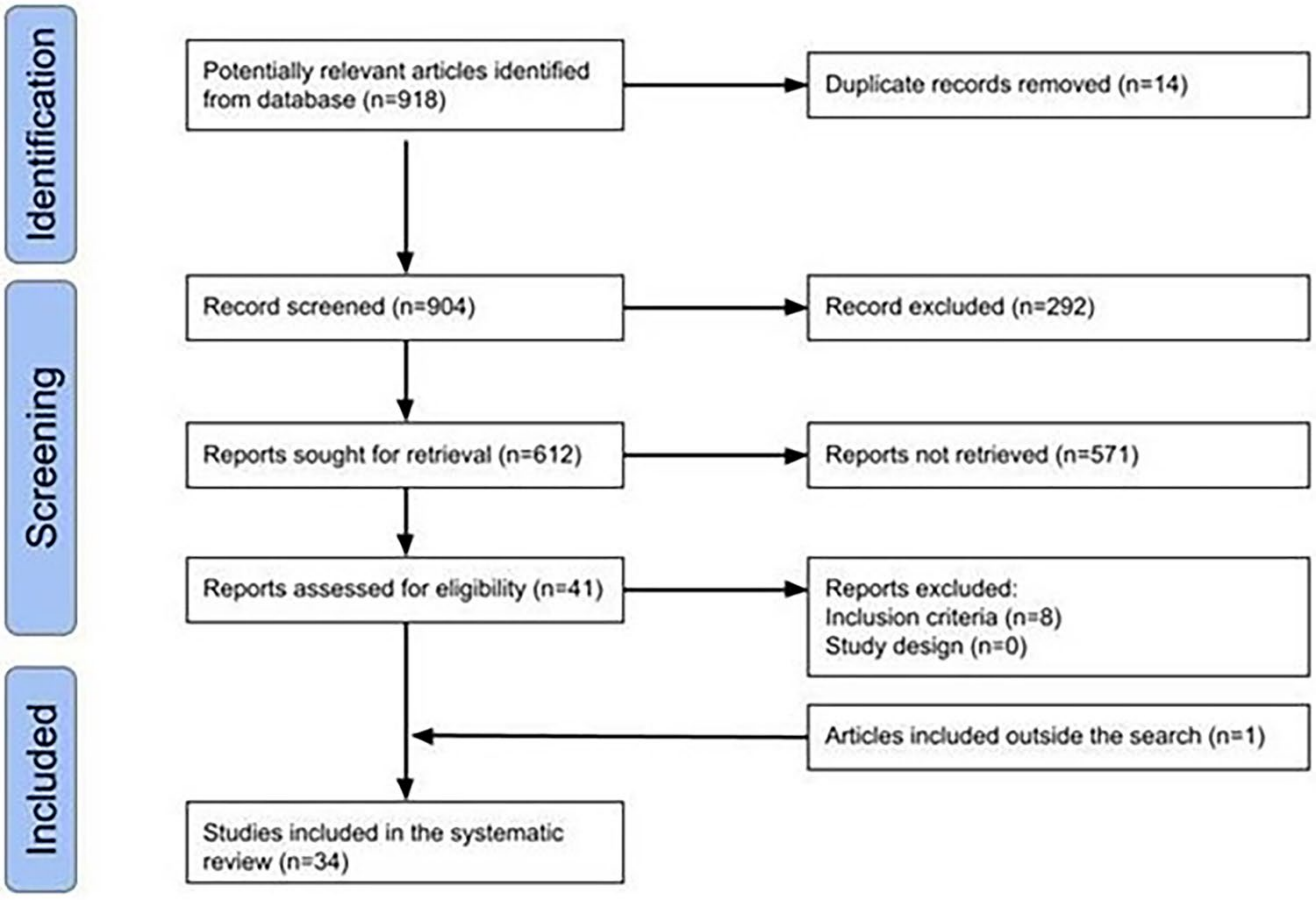
Flowchart

Using the search strategy, we found 918 studies. After carefully reviewing full-text studies, we finally included 34 studies (Fig. 1) [1, 3, 10–41].

### Characteristics of included studies

We included 1,208 mice and rats in the 34 studies for analysis. 83.44% of the population was Sprague Dawley rats, 6.29% was Wistar rats, 1.99% was Wistar Kyoto rats, 2.32% was Zucker rats, and 5.96% was mice. All studies involved animal experiments and included a control group free of erectile dysfunction and an intervention group that underwent various treatments or biomarker measurements. A total of 12 biomarkers were reviewed, distributed according to the number of studies as follows: eNOS in 28 studies (82.35%); VEGF in 6 studies (17.65%); nNOS and aSMA in 2 studies (5.88%); and FACL-4, PACS-2, IP3R1, endocan, endothelial microparticles, endothelial progenitor cells, LncRNA, and Hebp1 in at least one study each (2.94%).

Five detection methods were used: western blot in 27 studies (79.41%); immunofluorescence in 4 studies (11.76%); immunohistochemistry in 4 studies (11.76%); qRT-PCR in 3 studies (8.82%); and flow cytometry in 1 study (2.94%).

Depending on the type of marker, more than one penile tissue could be used for measurement. The most commonly used tissue was the corpus cavernosum in 30 studies (88.24%), the dorsal nerve of the penis was used in 2 studies (5.88%), the dorsal artery of the penis in 1 study (2.94%), and there were five studies in which the type of penile tissue used was not specified (14.71%) (Table 1).

**Table 1:** Characteristics of included studies

Author, year	Country	Species	Type of ED	Sample size	Biomarker	Method for measuring ED	Tissue	Detection methodology
Ryu JK, 2012	South Korea	Mice	Diabetic	16	eNOS	Intracavernous pressure	Corpus cavernosum	Western blot
Kim SJ,2012	South Korea	Rats: Sprague Dawley	Nerve injury	40	eNOS	Intracavernous pressure	Corpus cavernosum	Western blot
Liu G, 2013	China	Rats: Sprague Dawley	Diabetic	60	eNOS	Mean arterial pressure and Intracavernous pressure	Corpus cavernosum	Western blot
					VEGF		Corpus cavernosum	Western blot
Ouyang B, 2014	China	Rats: Sprague Dawley	Diabetic	65	VEGF	Mean arterial pressure and Intracavernous pressure	Corpus cavernosum	Western blot
Ryu JK,2015	South Korea	Mice	Dyslipidemia	24	eNOS	Intracavernous pressure	Corpus cavernosum	Western blot
Liao CH,2015	Taiwan	Rats: Sprague Dawley	Nerve injury	30	nNOS	Intracavernous pressure	Dorsal penile nerve	Immunofluorescence
					aSMA		Dorsal artery of the penis	Immunofluorescence
Jeon SH, 2016	South Korea	Rats: Sprague Dawley	Diabetic	50	nNOS	Intracavernous pressure	Corpus cavernosum	Western blot
					VEGF			
Jang H, 2017	South Korea	Rats: Sprague Dawley	Dyslipidemia	36	eNOS	Mean arterial pressure and Intracavernous pressure	Corpus cavernosum	Western blot
							Dorsal penile nerve	
Li R, 2017	China	Rats: Sprague Dawley	Dyslipidemia	70	eNOS	Mean arterial pressure and Intracavernous pressure	Corpus cavernosum	Western blot
Yin GN, 2020	South Korea	Mice	Nerve injury	12	eNOS	Intracavernous pressure	Corpus cavernosum	Western blot
Chiangsaen P, 2020	Thailand	Rats: Sprague Dawley	Hypertension	40	eNOS	Mean arterial pressure and Intracavernous pressure	Penile tissue	Western blot
Demirtaş ŞT, 2018	Turkey	Rats: Wistar	Stress and psychologic	16	eNOS	-	Corpus cavernosum	Immunohistochemistry
Seo D.Y, 2018	South Korea	Rats: Sprague Dawley	Stress and psychologic	28	eNOS	Intracavernous pressure	Corpus cavernosum	Western blot
Wang H, 2018	China	Rats: Sprague Dawley	Nerve injury	21	lncRNA MIAT	Mean arterial pressure and Intracavernous pressure	Penile tissue	qRT-PCR
Siddiquee AA, 2018	Singapore	Rats: Zucker	Diabetic	28	eNOS	Intracavernous pressure	Penile tissue	Western blot
Yazir Y, 2018	Turkey	Rats: Wistar	Stress and psychologic	32	eNOS	-	Corpus cavernosum	Immunohistochemistry
Jeon SH, 2018	South Korea	Rats: Sprague Dawley	Diabetic	48	eNOS	Mean arterial pressure and Intracavernous pressure	Corpus cavernosum	Western blot
Jung AR, 2019	South Korea	Rats: Sprague Dawley	Nerve injury	28	eNOS	Mean arterial pressure and Intracavernous pressure	Corpus cavernosum	Western blot
					aSMA			Immunohistochemistry
Chen S, 2019	China	Rats: Sprague Dawley	Diabetic	40	eNOS	Mean arterial pressure and Intracavernous pressure	Corpus cavernosum	Immunofluorescence
Huang T, 2019	China	Rats: Sprague Dawley	Chronic prostatitis	40	eNOS	Mean arterial pressure and Intracavernous pressure	Corpus cavernosum	Western blot
Song J, 2020	China	Rats: Sprague Dawley	Diabetic	41	eNOS	Mean arterial pressure and Intracavernous pressure	Penile tissue	Western blot
Mukti A.I, 2021	Indonesia	Rats: Sprague Dawley	Nerve injury	24	eNOS	-	Corpus cavernosum	Immunohistochemistry
					VEGF			qRT-PCR
Oztekin CV, 2021	Turkey	Rats: Sprague Dawley	Diabetic	25	eNOS	-	Penile tissue	Western blot
Lee J, 2021	South Korea	Rats: Sprague Dawley	Nerve injury	70	eNOS	Mean arterial pressure and Intracavernous pressure	Corpus cavernosum	Western blot
Li X, 2021	China	Rats: Wistar-Kyoto	Hypertension	24	Endothelial microparticles	Mean arterial pressure and Intracavernous pressure	Corpus cavernosum	Flow cytometry
					Endothelial progenitor cells			
Yin GN, 2022	South Korea	Mice	Diabetic	20	Hebp1	Intracavernous pressure	Corpus cavernosum	Western blot
								Immunofluorescence
Wu JH, 2022	China	Rats: Sprague Dawley	Diabetic	30	eNOS	Intracavernous pressure	Corpus cavernosum	qRT-PCR
					VEGF		Corpus cavernosum	
Wang S, 2022	China	Rats: Wistar	Diabetic	28	VEGF	Mean arterial pressure and Intracavernous pressure	Corpus cavernosum	Western blot
					eNOS			
Yang HZ,2022	China	Rats: Sprague Dawley	Hypogonadism	36	eNOS	Mean arterial pressure and Intracavernous pressure	Corpus cavernosum	Western blot
					FACL-4			
					PACS-2			
					IP3R1			
Gu Q, 2024	China	Rats: Sprague Dawley	Chronic prostatitis	30	eNOS	Mean arterial pressure and Intracavernous pressure	Corpus cavernosum	Western blot
Chen Z, 2024	China	Rats: Sprague Dawley	Hypogonadism	36	eNOS	Mean arterial pressure and Intracavernous pressure	Corpus cavernosum	Western blot
					Endocan			
Mao Y, 2024	China	Rats: Sprague Dawley	Diabetic	30	eNOS	Mean arterial pressure and Intracavernous pressure	Corpus cavernosum	Western blot
Luo L, 2024	China	Rats: Sprague Dawley	Diabetic	60	eNOS	Mean arterial pressure and Intracavernous pressure	Corpus cavernosum	Western blot

### Characteristics of excluded studies

The exclusion criteria were the minimum number of reported individuals, the full text being unavailable, a population not meeting the inclusion criteria, the same study design, or a study in which the primary outcome was not measured.

### Risk of bias assessment

**Fig. 2:**
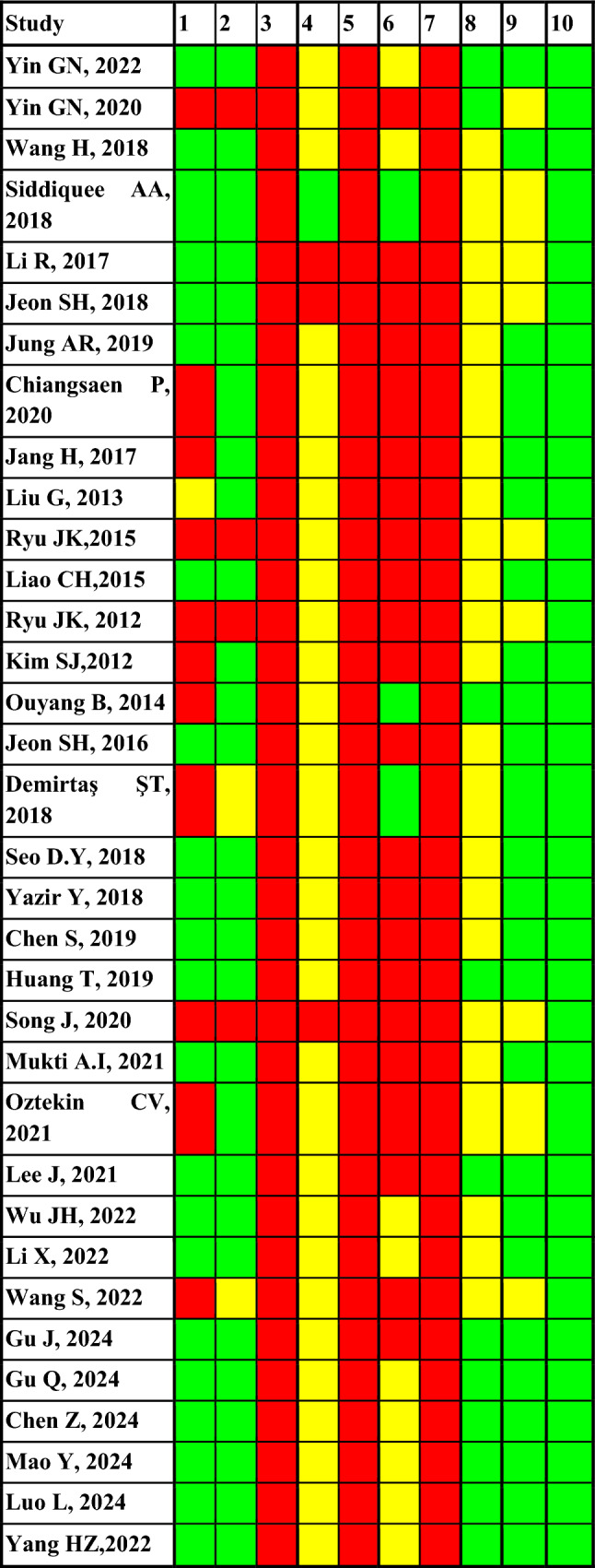
Risk of bias assessment

Selection, performance, and detection biases were identified in 100% of the studies. In 67% of studies, the loss of information or attrition due to death or other causes is not clarified. In 26% of studies, there is a probable risk of reporting bias. No other bias was identified in the different studies included. The highest risk of bias was identified in the following studies: Yin GN et al. 2020; Ryu JK et al. 2015; Ryu JK et al. 2012; Song J et al. 2020; Wang S et al. 2022 (Fig. 2).

### Primary outcomes

The results are presented for each of the biomarkers, with a comparative analysis between the control group (healthy control) and the intervention group (erectile dysfunction). The relative expression proportions for each group are shown, along with the number of individuals for whom the relative proportion is expressed; this value is expressed as n. The *p* value is represented for each study, all statistically significant except for the Oztekin CV et al. 2021 study. (Tables 2, 3).

**Table 2:** Markers with decreased expression

Biomarker	Author, year	Total group	Control group	Relative ratio (n) *	Intervention group	Relative ratio	Proportion (%)	Statistical significance
eNOS	Ryu JK, 2012	16	4	1.0 (4)	12	0.55	− 45	*p* < 0.01
	Kim SJ,2012	40	10	0.7 (10)	30	0.2	− 71.43	*p* < 0.05
	Liu G, 2013	60	12	0.75 (12)	48	0.1	− 86.67	*p* < 0.05
	Ryu JK,2015	24	4	1.0 (4)	20	0.33	− 67	*p* < 0.01
	Jang H, 2017	36	12	0.5 (12)	24	0.27	− 46	*p* < 0.01
	Li R, 2017	70	8	0.5 (5–7)	62	0.3	− 40	*p* < 0.05
	Siddiquee AA, 2018	28	7	1.8 (7)	21	0.7	− 61.11	*p* < 0.05
	Jeon SH, 2018	48	12	0.35 (8)	36	0.12	− 65.71	*p* < 0.05
	Demirtaş ŞT, 2018	16	8	1.9 (8)	8	0.5	− 73.68	*p* < 0.05
	Seo D.Y, 2018	28	7	1.0 (7)	21	0.8	− 20	*p* < 0.05
	Yazir Y, 2018	32	8	1.0 (8)	24	0.25	− 75	*p* < 0.01
	Chen S, 2019	40	10	1.0 (5)	30	0.2	− 80	*p* < 0.05
	Huang T, 2019	40	20	0.22 (20)	20	0.08	− 36.36	*p* < 0.05
	Jung AR, 2019	28	7	0.8 (7)	21	0.2	− 75	*p* < 0.05
	Song J, 2020	41	10	0.7 (10)	31	0.25	− 64.29	*p* < 0.05
	Yin GN, 2020	12	3	1.0 (3)	12	0.1	− 90	*p* < 0.05
	Chiangsaen P, 2020	40	8	1.0 (4)	32	0.5	− 50	*p* < 0.05
	Oztekin CV, 2021 **	25	5	0.8 (5)	20	0.9	+ 12.5	*p* > 0.05
	Lee J, 2021	70	14	1.0 (14)	56	0.4	− 60	*p* < 0.05
	Mukti A.I, 2021	24	6	0.23 (6)	18	0.16	− 30.43	*p* < 0.05
	Wu JH, 2022	30	10	1.0 (10)	20	1.0	0	*p* < 0.05
	Wang S, 2022	28	10	0.38 (10)	18	0.08	− 78.95	*p* < 0.05
	Yang HZ,2022	36	6	0.7 (6)	30	0.3	− 57.14	*p* < 0.01
	Gu J, 2024	30	6	0.41 (6)	24	0.18	− 56.1	*p* < 0.01
	Gu Q, 2024	30	15	1.0 (3)	15	0.6	− 40	*p* < 0.01
	Mao Y, 2024	30	6	1.0 (6)	24	0.45	− 55	*p* < 0.01
	Luo L, 2024	60	10	1.0 (10)	50	0.7	− 30	*p* < 0.01
	Chen Z, 2024	36	6	1.0 (6)	30	0.74	− 26	*p* < 0.05
VEGF	Liu G, 2013	60	12	1.3 (12)	48	0.1	− 92.31	*p* < 0.05
	Ouyang B, 2014	65	10	1.7 (10)	55	0.4	− 76.47	*p* < 0.05
	Jeon SH, 2016	50	10	0.62 (10)	40	0.26	− 58.06	*p* < 0.05
	Mukti A.I, 2021	24	6	2.0 (6)	18	1.0	− 50	*p* < 0.05
	Wu JH, 2022	30	10	2.0 (10)	20	1.0	− 50	*p* < 0.01
	Wang S, 2022	28	10	0.5 (10)	18	0.25	− 50	*p* < 0.05
nNOS	Jeon SH, 2016	50	10	0.42 (10)	40	0.18	− 57.14	*p* < 0.05
	Liao CH,2015	30	8	0.35 (8)	24	0.12	− 65.71	*p* < 0.01
aSMA	Liao CH,2015	30	8	0.14 (8)	24	0.07	− 50	*p* < 0.05
	Jung AR, 2019	28	7	0.17 (7)	21	0.05	− 70.59	*p* < 0.05
lncRNA MIAT	Wang H, 2018	21	7	1.0 (7)	14	0.5	− 50	*p* < 0.05
Hebp1	Yin GN, 2022	20	5	1.0 (4)	15	0.4	− 60	*p* < 0.001

**Table 3:** Markers with increased expression

Biomarker	Author, year	Total group	Control group	Relative ratio (n) *	Intervention group	Relative ratio	Proportion (%)	Statistical significance
FACL-4	Yang HZ,2022	36	6	0.3 (6)	30	0.5	+ 66.67	*p* < 0.01
PACS-2	Yang HZ,2022	36	6	0.21 (6)	30	0.55	+ 161.9	*p* < 0.01
IP3R1	Yang HZ,2022	36	6	0.09 (6)	30	0.2	+ 122.2	*p* < 0.01
Endocan	Chen Z, 2024	36	6	0.43 (6)	30	1.0	+ 132.56	*p* < 0.05
Endothelial microparticles	Li X, 2022	24	6	0.9 (6)	18	1.6	+ 77.78	*p* < 0.05
Endothelial progenitor cells	Li X, 2022	24	6	1.5 (6)	18	2.7	+ 80	*p* < 0.05

### Markers with decreased expression (Table 2)

#### eNOS

Ten studies showed a decline of more than 50% [13, 14, 17–19, 21, 22, 24, 25, 42]. Only two studies showed no decrease [28, 31]. Nine studies showed a reduction of less than 50% [1, 11, 15, 16, 20, 23, 30, 34, 36].

#### VEGF

All studies showed a decrease of more than 50%. [13, 18, 30–32, 37].

#### nNOS

The total number of studies showed a decrease of more than 50% [18, 39].

#### aSMA

All studies showed a decrease of more than 50% [24, 39].

#### LncRNA

One study showed a decrease greater than 50% [40].

#### Hebp1

One study showed a decrease greater than 50% [41].

### Markers with increased expression (Table 3)

#### FACL-4

One study showed an increase of more than 50 [3].

#### PACS-2

One study showed an increase of more than 50% [3].

#### IP3R1

One study showed an increase of more than 50% [3].

#### Endocan

One study showed an increase of more than 50% [1].

#### Endothelial microparticles

One study showed an increase of more than 50% [10].

#### Endothelial progenitor cells

One study showed an increase of more than 50% [10].

## Discussion

Endothelial damage has been documented as a possible precursor of erectile dysfunction; in turn, it may have markers indicating the presence of such endothelial dysfunction [2, 5, 9, 10]. There is a review by *Kim SW *et al*.* in rats with induced diabetes mellitus and treatments with mesenchymal cells administered for erectile dysfunction, where the pathways potentially involved in the treatments included VEGF, PECAM, vWF, nNOS, SDF-1, eNOS, and Bcl-2 [42]. However, this review did not focus on identifying variations in marker levels between control and intervention groups but instead on the outcome of improvement in erectile dysfunction.

Our systematic review is the first to be based on preclinical studies and to consider multiple reported markers. The sample size in each study is variable, but the number of studies for some markers is high, as seen with eNOS, which was measured in 28 studies. We found that six markers showed a decrease in expression, and six showed an increase.

It can be observed that for all markers (eNOS, VEGF, nNOS, aSMA, LncRNA, Hebp1) with decreased expression due to endothelial damage, most studies showed a reduction of more than 50% compared to control groups. This differs from the literature; for example, *Song Km *et al*.* showed that eNOS expression was reduced by more than 50%, but this was not the case for VEGF [43]. There is a meta-analysis in humans regarding endocan in diabetic patients with or without other comorbidities; endocan levels were significantly higher in those with ED, with a mean difference of 0.94 (95% CI: 0.20–1.67; P < 0.01). This finding is consistent with the results of our systematic review in preclinical trials [44].

Among the limitations of our study is the heterogeneity of the rodent populations used (five rat strains and mice), as well as the use of different diagnostic methods (western blot, immunofluorescence, immunohistochemistry, qRT-PCR, and flow cytometry). In addition, each study's control and/or intervention groups varied in their participant populations. The cutoff point for the relative rate in the control group was arbitrarily established in each study, which made it difficult to calculate the percentage variability of the marker when comparing increases or decreases between intervention and control groups. This could lead to information bias in our study, which is consistent with the generalized risk of bias observed in the included preclinical trials.

Regarding the risk of bias implications, a high risk of selection, performance, and detection biases was observed across all included studies, reflecting common methodological limitations in preclinical research. The overall internal validity was therefore limited, particularly in studies such as Yin GN et al. 2020; Ryu JK et al. 2012, 2015; Song J et al. 2020; and Wang S et al. 2022. These findings underscore the need for stricter adherence to reporting guidelines like ARRIVE and SYRCLE to enhance transparency and reproducibility in future animal research.

One of the advantages is the use of induced scenarios in rodents, such as hypogonadism, diabetes mellitus, obesity, etc.; these latter conditions are closely related to metabolic syndrome, which leads to cardiovascular disease or a microvascular environment that results in erectile dysfunction in humans. Likewise, nerve injury to the penis was induced in the animals, which could occur in scenarios such as radical prostatectomy.

One important consideration is that the tissue used for measuring endothelial damage was taken directly from the penis of the animals. In future, these findings could allow for systemic diagnosis tests that correlate with microvascular penile endothelial damage without the need for invasive diagnostic methods, potentially enabling earlier and more accurate prediction of erectile dysfunction.

In future, clinical studies could focus on evaluating the diagnostic performance of the different measurements and, at the same time, determine the accuracy needed to establish these tests as part of a standard model for diagnosing endothelial damage.

## Conclusions

eNOS; VEGF; nNOS; aSMA; LncRNA; and Hebp1 showed decreased expression in the presence of endothelial damage, while FACL-4; PACS-2; IP3R1; endocan; endothelial microparticles; and endothelial progenitor cells showed an increase under the same conditions and could become markers with implications in clinical trials and the development of new targeted treatments for ED.

## Data Availability

No datasets were generated or analyzed during the current study.

## Abbreviations

**ED**: Erectile dysfunction
**AKT**: Protein kinase B
**eNOS**: Endothelial nitric oxide synthase
**NO**: Nitric oxide
**nNOS**: Neuronal nitric oxide synthase
**ESM-1**: Endothelial cell-specific molecule 1 (endocan)
**FACL-4**: Fatty acid-CoA ligase 4
**PACS-2**: Phosphofurin acidic cluster sorting protein 2
**IP3R1**: Inositol 1,4,5-trisphosphate receptor type 1
**HIF-1****α**: Hypoxia-inducible factor 1 alpha
**PDE5**: Phosphodiesterase type 5
**HUCB-MNCs**: Human umbilical cord blood-derived mononuclear cells
**UC-MSCs**: Umbilical cord-derived mesenchymal stem cells
**DMED**: Diabetes mellitus-induced erectile dysfunction
**STZ**: Streptozotocin
**PBS**: Phosphate-buffered saline
**APO**: Apomorphine
**DMEM**: Dulbecco’s modified Eagle medium
**FBS**: Fetal bovine serum
**UCMS**: Unpredictable chronic mild stress
**ETN**: Etanercept
**CC**: Corpus cavernosum
